# Neuroprotective effects of melittin on hydrogen peroxide-induced apoptotic cell death in neuroblastoma SH-SY5Y cells

**DOI:** 10.1186/1472-6882-14-286

**Published:** 2014-08-05

**Authors:** Sang Mi Han, Jung Min Kim, Kwan Kyu Park, Young Chae Chang, Sok Cheon Pak

**Affiliations:** Department of Agricultural Biology, National Academy of Agricultural Science, RDA, Suwon, 441-100 Korea; College of Medicine, Catholic University of Daegu, Daegu, 712-702 Korea; School of Biomedical Sciences, Charles Sturt University, Bathurst, NSW 2795 Australia

## Abstract

**Background:**

Free radicals are involved in neuronal cell death in human neurodegenerative diseases. Since ancient times, honeybee venom has been used in a complementary medicine to treat various diseases and neurologic disorders. Melittin, the main component of honeybee venom, has various biologic effects, including anti-bacterial, anti-viral, and anti-inflammatory activities.

**Methods:**

We investigated the neuroprotective effects of melittin against H_2_O_2_-induced apoptosis in the human neuroblastoma cell line SH-SY5Y. The neuroprotective effects of melittin on H_2_O_2_-induced apoptosis were investigated using a 3-(4,5-dimethylthiazol-2-yl)-2,5-diphenylterazolium bromide assay, caspase 3 activity, 4,6-diamidino-2-phenylindole staining, a lactate dehydrogenase release assay, Western blots, and reverse transcription-polymerase chain reaction.

**Results:**

The H_2_O_2_-treated cells had decreased cell viability with apoptotic features and increased production of caspase-3. On the other hand, melittin treatment increased cell viability and decreased apoptotic DNA fragmentation. Melittin attenuated the H_2_O_2_-induced decrease in mRNA and protein production of the anti-apoptotic factor Bcl-2. In addition, melittin inhibited both the H_2_O_2_-induced mRNA and protein expression of Bax-associated pro-apoptotic factor and caspase-3.

**Conclusions:**

These findings suggest that melittin has potential therapeutic effects as an agent for the prevention of neurodegenerative diseases.

## Background

Oxidative stress is implicated as a causative factor in neurodegenerative diseases, including Alzheimer’s disease, Parkinson’s disease, Huntington’s disease, and amyotrophic lateral sclerosis [1–3]. Reactive oxygen species (ROS), such as superoxide anions, hydroxyl radicals, and hydrogen peroxide (H_2_O_2_), are easily generated in redox processes that occur in the human body. These ROS induce oxidative stress, which can cause dysfunction of mitochondria, proteins, DNA and lipid membranes, and eventually disrupt cellular function and integrity [4–7]. Among the various ROS, H_2_O_2_ induces apoptosis in a variety of cells and acts as a precursor of ROS [8]. In addition, H_2_O_2_ diffuses easily in and out of cells and tissues [9]. H_2_O_2_-induced apoptosis is regulated by the activation of Bcl-2 family members [10]. Upregulation of the pro-apoptotic enzyme Bax and the downregulation of the anti-apoptotic enzyme Bcl-2 both induce cell apoptosis, which could interfere with the execution phases of apoptosis, including the caspase pathway [11, 12].

Melittin, the major bioactive component of honeybee venom (*Apis mellifera*), is a cationic, hemolytic peptide comprising a small linear peptide composed of 26 amino acid residues. The amino-terminal region is hydrophobic, and the carboxyl-terminal region is hydrophilic [13, 14]. Previous studies demonstrated that melittin has anti-bacterial [14], anti-arthritic [15], and anti-inflammatory [16] effects in various cell lines. Melittin also has anti-apoptotic effects by activating Bcl-2 and suppressing Bax and caspase 3 in transforming growth factor (TGF)-β1-induced injury to hepatocytes [17]. In addition, melittin inhibits caspase and Bax expression in D-galactosamine/lipopolysaccharide induced acute hepatic failure [18]. The mechanisms of the neuroprotective effects of melittin in H_2_O_2_-induced neuroblastoma SH-SY5Y cells, however, have not been fully elucidated.

In the present study, we investigated whether melittin protects against H_2_O_2_-induced neurotoxicity and explored the possible mechanisms of action by examining the upregulation of the anti-apoptotic enzyme Bcl-2 and the downregulation of the pro-apoptotic enzymes Bax and caspase 3.

## Methods

### Materials

Dulbecco’s modified Eagle’s medium, fetal bovine serum, and penicillin/streptomycin (100 IU/50 μg/ml) were obtained from Invitrogen (Grand Island, NY). Mellitin, H_2_O_2_, 3-(4,5-dimethylthizaol-2-yl)-2,5-diphenyltetrazolium bromide (MTT), dimethyl sulfoxide, 4′, 6-diamidino-2-phenylindole (DAPI), 2′,7′-dichlorofluorescein diacetate, rabbit anti-Bax, rabbit anti-Bcl-2, and rabbit anti-caspase-3 were purchased from Abcam (Cambridge, MA). Anti-rabbit horseradish peroxidase-linked IgG antibodies were purchased from GE Healthcare Life Science (Buckinghamshire, England, UK). All other chemicals were of analytical grade.

### Cell culture and treatment

Human neuroblastoma SH-SY5Y cells, obtained from the Korea Cell Line Bank (Seoul, Korea), were cultured in Dulbecco’s modified Eagle’s medium supplemented with 10% (v/v) fetal bovine serum, and 1% penicillin/streptomycin at 37°C under 5% CO_2_ in air. To determine the effect of melittin on H_2_O_2_-exposed SH-SY5Y cells, SH-SY5Y cells were treated with various doses of melittin for 1 h before H_2_O_2_ exposure for 6 h. H_2_O_2_ was prepared immediately before use as a 20 mM stock. Melittin was dissolved in phosphate-buffered saline (PBS) and the stock solutions were added directly to the culture media. In a single experiment, each treatment was performed in triplicate.

### Cell viability assay

Cell viability was determined by MTT assay. SH-SY5Y cells were seeded in 96-well plates at density of 8 × 10^4^ cells/well and incubated for 48 h prior to experimental treatments. The cells were pre-incubated with or without melittin following incubation with H_2_O_2_ for 24 h. The cultured medium was removed and 50 μl MTT solution (1 mg/ml in PBS) was placed in each well. After incubation at 37°C for 4 h, the solution was carefully removed, and 150 μl dimethyl sulfoxide was added. Absorbance was measured at 570 nm using a microplate reader (Bio-Tek Instruments, Inc., Winooski, VT).

### Lactate dehydrogenase release assay

Lactate dehydrogenase (LDH) is released into the cell culture supernatant when cells undergo by apoptosis or necrosis. LDH levels were measured using a Cytotoxicity Cell Death kit (Takara Bio, Shiga, Japan) according to the manufacturer’s instructions. Briefly, the cells (8 × 10^4^ cells/well) were seeded in 96-well plates and then incubated with 100 μM H_2_O_2_ for 24 h with or without melittin pretreatment for 1 h. For analysis, 100 μl supernatant was transferred to a new 96 well plate, and 100 μl of reaction mixture was added to each well and incubated at 37°C for 30 min. Absorbance was measured at 490 nm using microplate reader (Bio-Tek Instruments, Inc.). LDH release was determined in cells treated with 2% Triton X-100 (high control); the assay medium served as the low control and was subtracted from all absorbance measurements.

### Nuclear staining with DAPI

Nuclear morphology was assessed by staining with DAPI. Cells (1 × 10^5^ cells/well) were seeded on coverslips in 6-well plates for 48 h and then treated with 100 μM of H_2_O_2_ for 24 h with or without melittin pretreatment for 1 h. The cells were washed twice with PBS and fixed with 1% paraformaldehyde for 15 min. The fixed cells were washed twice with PBS and stained with 4′, 6-DAPI (1 μg/ml) for 10 min at 37°C in the dark. Cells were washed twice with PBS and were observed using a fluorescent microscope.

### Caspase-3 activity

Activation of caspase-3 was determined according to the protocols recommended for the caspase-3 assay kit (R&D Systems, Minneapolis, MN). In brief, the cells were lysed and centrifuged to obtain the supernatant. The supernatant was added to the reaction mixture containing dithiothreitol and caspase-3 substrate, and incubated for 2 h at 37°C. Absorbance was measured at a wavelength of 405 nm using a microplate reader (Bio-Tek Instruments, Inc.).

### Western blot analysis

Cell were lysed with ice-cold lysis buffer containing protease inhibitors and centrifuged at 14,000 rpm for 10 min. The protein content of each supernatant was determined using a Bradford assay with bovine serum albumin as the protein standard. Samples (10 μg) were separated by polyacrylamide gel (10%) electrophoresis, and then transferred to a polyvinylidene difluoride membrane (0.45 μm, Immobilon-P Transfer membrane, Millipore, Billerica, MA). The membranes were blocked with 5% non-fat dry milk in Tris-buffered saline containing 0.1% Tween 20 (TBST) for 1 h. After blocking, membranes were incubated with Bcl-2, Bax, and caspase-3 antibody (Abcam) in TBST overnight at 4°C. After washing in TBST, the membranes were incubated with horseradish peroxidase-conjugated secondary antibodies (GE Healthcare Life Science) at a 1:5000 dilution for 1 h at room temperature. After washing with TBST, proteins were visualized using a Super Signal West Pico Kit (Pierce, Rockford, IL) detection system. Densitometric analysis was performed using Quantity One (Bio-Rad, Hercules, CA) to scan the signals.

### RNA extraction and reverse transcription-polymerase chain reaction

Total RNA was isolated using the Total RNA Purification kit (Nanohelix, Daejeon, Korea) according to the manufacturer’s instructions. Reverse transcription of total RNA (1 μg) was performed for 1 h at 45°C using RT Premix kit (Oligo dT primer; iNtRON Biotechnology, Sungnam, Korea). The reaction was terminated by heating at 95°C at 5 min. cDNA was amplified by polymerase chain reaction (PCR) Premix kit (i-Taq) (iNtRON Biotechnology). Sequences of primers for Bcl-2 cDAN are: forward primer 5′-CGACTTCGCCGAGATGTCCAGCCAG-3′ and reverse primer 5′-ACTTGTGGCCCAGATAGGCACCCAG-3′, for Bax cDNA are: forward primer 5′-ACCAAGAAGCTGAGCGAGTGTC-3′ and reverse primer 5′-TGTCCAGCCCATGATGGTTC-3′, and for glyceraldehyde-3-phosphate dehydrogenase (GAPDH) cDNA are: forward primer 5′-AATGACCCCTTCATTGAC-3′ and reverse primer 5′-TCCACGACGTACTCAGCGC-3′. PCR for Bcl-2 and GAPDH) [19] was performed with 35 cycles as follows: denaturation at 94°C for 30 s, annealing at 61°C for 1 min, and extension at 72°C for 1 min. PCR for Bax [20] was performed with 40 cycles and the reaction conditions were: denaturation at 94°C for 45 s, annealing at 61°C for 1 min, and extension at 72°C for 1 min using PCR Thermal Cycler Dice (Takara, Shiga, Japan). The PCR products were analyzed by 2% agarose gel electrophoresis with ethidium bromide. The signal intensity of each band was quantified and normalized against GAPDH. Densitometric analysis was carried out using Quantity One (Bio-Rad) to scan the signals.

### Statistical analysis

All data are expressed as the mean ± standard error of the mean (SEM). Statistical differences among groups were calculated by analysis of variance (ANOVA) followed by Duncan’s multiple range test (SPSS version 18.0, Chicago, IL). Differences with a p value less than 0.05 were considered significant.

## Results

### Melittin inhibited H_2_O_2_-induced apoptosis of SH-SY5Y cells

**Figure 1:**
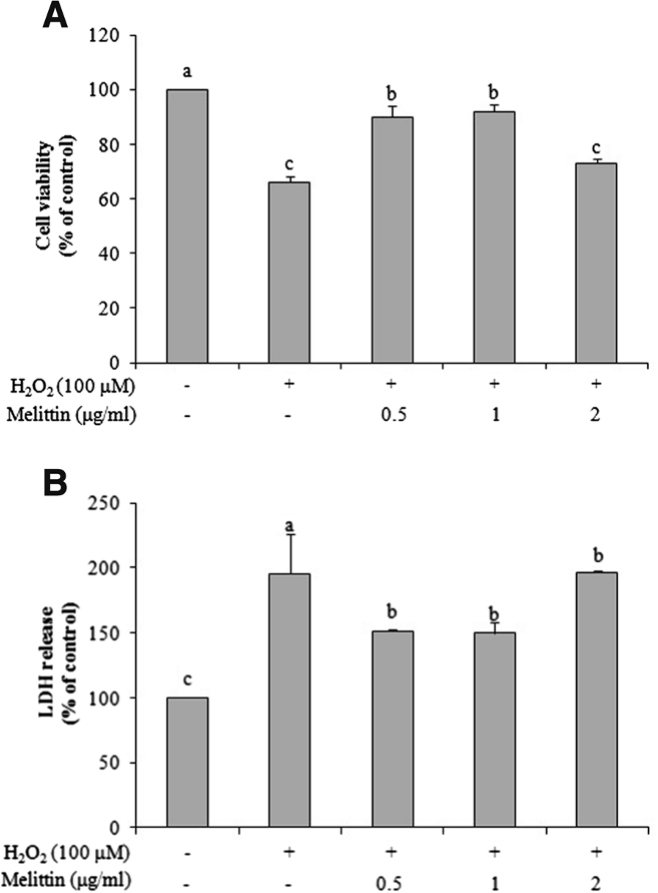
**Effect of melittin on H**_**2**_ **O**_**2**_ **-induced apoptosis in SH-SY5Y neuroblastoma cells. (A)** Melittin increased the cell viability of H_2_O_2_-treated SH-SY5Y cells as determined by MTT assay. **(B)** Melittin decreased LDH release as determined using a cytotoxicity detection kit. Data are expressed as mean ± SEM from three independent experiments. Bars with different letters indicate significant differences at p < 0.05.

We examined the protective effects of melittin against H_2_O_2_-induced apoptotic cell death of SH-SY5Y. SH-SY5Y cells were exposed to 100-400 μM H_2_O_2_ for 6 h. The lowest dose of H_2_O_2_ (100 μM) induced 66.0% cell viability compared with the control (data not shown) and was therefore used for the following experiments. SH-SY5Y cells were pretreated with various doses of melittin for 1 h, followed by 100 μM H_2_O_2_ for 6 h. In Figure 1A, the low doses of melittin (0.5 and 1 μg/ml) significantly attenuated the H_2_O_2_-induced cytotoxicity.

To further investigate the protective effect of melittin, we measured LDH release (Figure 1B). LDH release significantly increased after exposure with 100 μM H_2_O_2_ for 6 h. In contrast, the LDH release was decreased by pretreatment with 0.5 and 1 μg/ml melittin for 1 h compared to the H_2_O_2_ only treated group. In Figure 1B, the 2 μg/ml dose is also labeled as significantly different from the H_2_O_2_ group, although the data do not look significantly different. The higher dose of melittin (2 μg/ml), however, significantly increased LDH release. Therefore, only the 0.5 to 1 μg/ml doses of melittin were used for subsequent experiments.

### Melittin protected H_2_O_2_-induced morphological changes of SH-SY5Y cells

**Figure 2:**
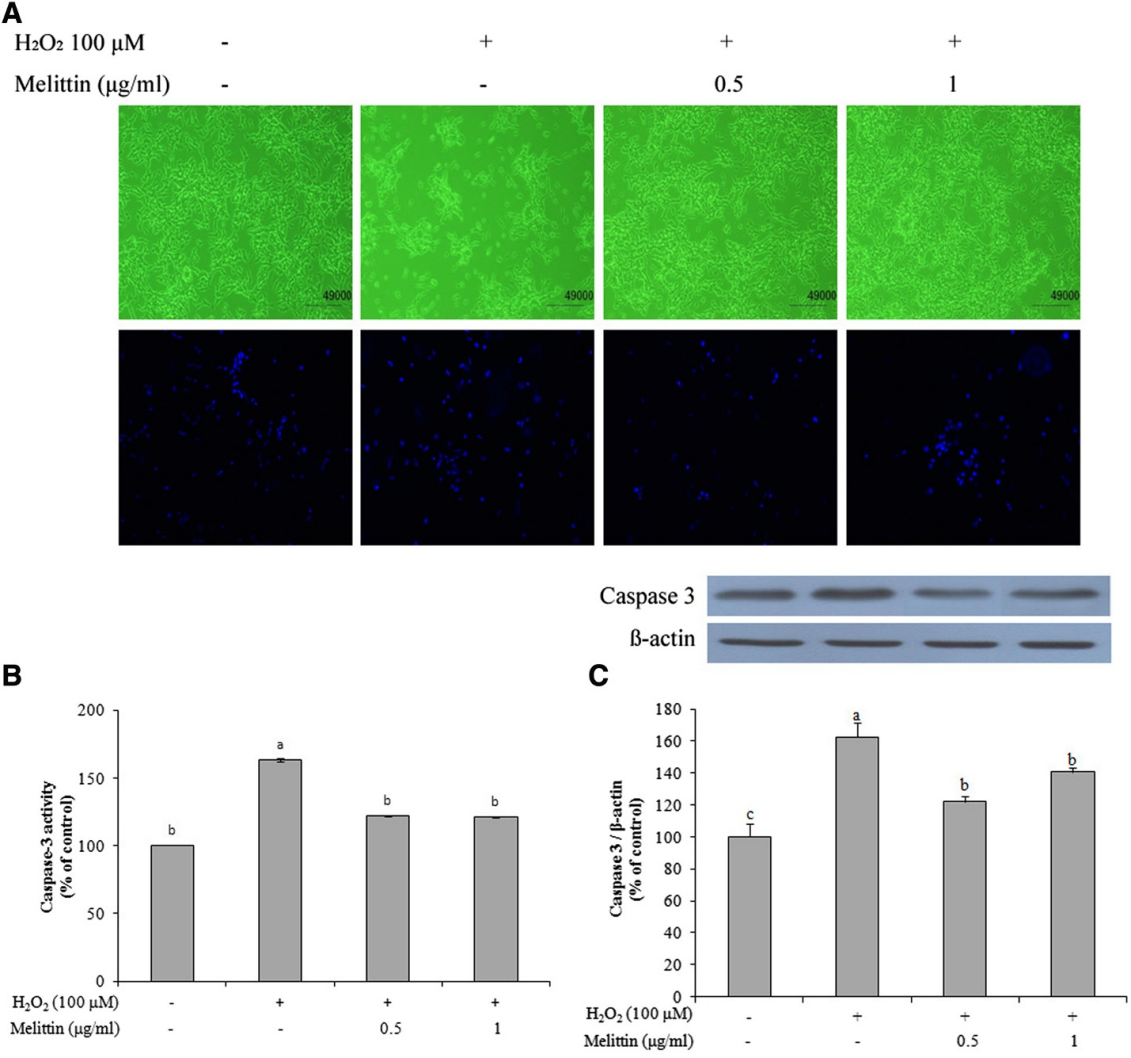
**Effect of melittin on nuclear morphology and caspase-3 activity in H**_**2**_ **O**_**2**_ **-treated SH-SY5Y cells. (A)** Morphology of SH-SY5Y cells treated with H_2_O_2_ in the absence or presence of melittin for 6 h. Representative nuclear morphology was determined by a fluorescent DNA-binding agent DAPI staining and visualized by a fluorescence microscope (10×). **(B)** Melittin attenuated H_2_O_2_-induced increase in caspase-3 activity as determined using a caspase colormetric assay kit. **(C)** Melittin suppressed H_2_O_2_-induced expression of caspase-3 protein as determined by Western blot analysis. Data are expressed as mean ± SEM from three independent experiments. Bars with different letters indicate significant differences at p < 0.05.

The protective effects of melittin were confirmed by morphological observations (Figure 2A). Morphologic changes were observed in 100 μM H_2_O_2_-treated SH-SY5Y cells. Pretreatment with melittin prevented the H_2_O_2_-induced morphologic changes. DAPI staining also showed nuclear condensation and DNA fragmentation following treatment with 100 μM H_2_O_2_. Pretreatment with melittin inhibited these apoptotic features.

### Melittin suppressed caspase-3 activation and expression of H_2_O_2_-treated SH-SY5Y cells

We investigated the protective effect of melittin on caspase-signaling. In H_2_O_2_-treated SH-SY5Y cells, caspase-3 activity was significantly increased by 63% of the control, but pretreatment with 0.5 and 1 μg/ml melittin attenuated the H_2_O_2_-induced increase in caspase-3 activity by approximately 41% and 42%, respectively (Figure 2B). In addition, we examined caspase-3 protein expression in H_2_O_2_ and/or melittin-treated SH-SY5Y cells by Western blotting. H_2_O_2_ treatment increased caspase-3 protein expression by 63% compared to that of control (Figure 2C). Pretreatment with melittin, however, suppressed the H_2_O_2_-induced expression of caspase 3 protein (0.5 μg/ml: 22%; 1 μg/ml: 41%) to level similar to the control.

### Melittin decreased the ratio of Bax/Bcl-2 of H_2_O_2_-treated SH-SY5Y cells

**Figure 3:**
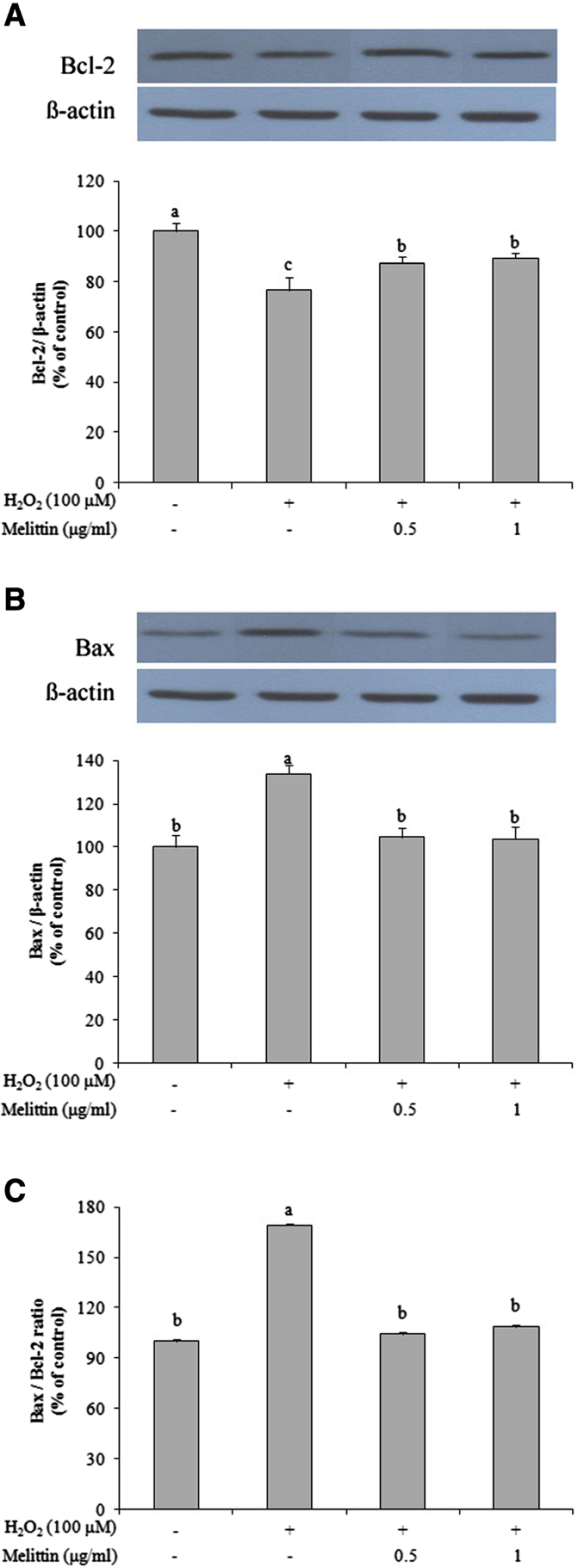
**Effect of melittin on protein expression of Bcl-2 and Bax in H**_**2**_ **O**_**2**_ **-treated SH-SY5Y cells. (A)** Melittin inhibited the decrease of Bcl-2 protein as measured by Western blot analysis. **(B)** Melittin blocked the increase of Bax protein as determined by Western blot analysis. **(C)** Melittin caused a low Bax/Bcl-2 protein ratio. Data are expressed as mean ± SEM from 3 independent experiments. Bars with different letters indicate significant differences at p < 0.05.

**Figure 4:**
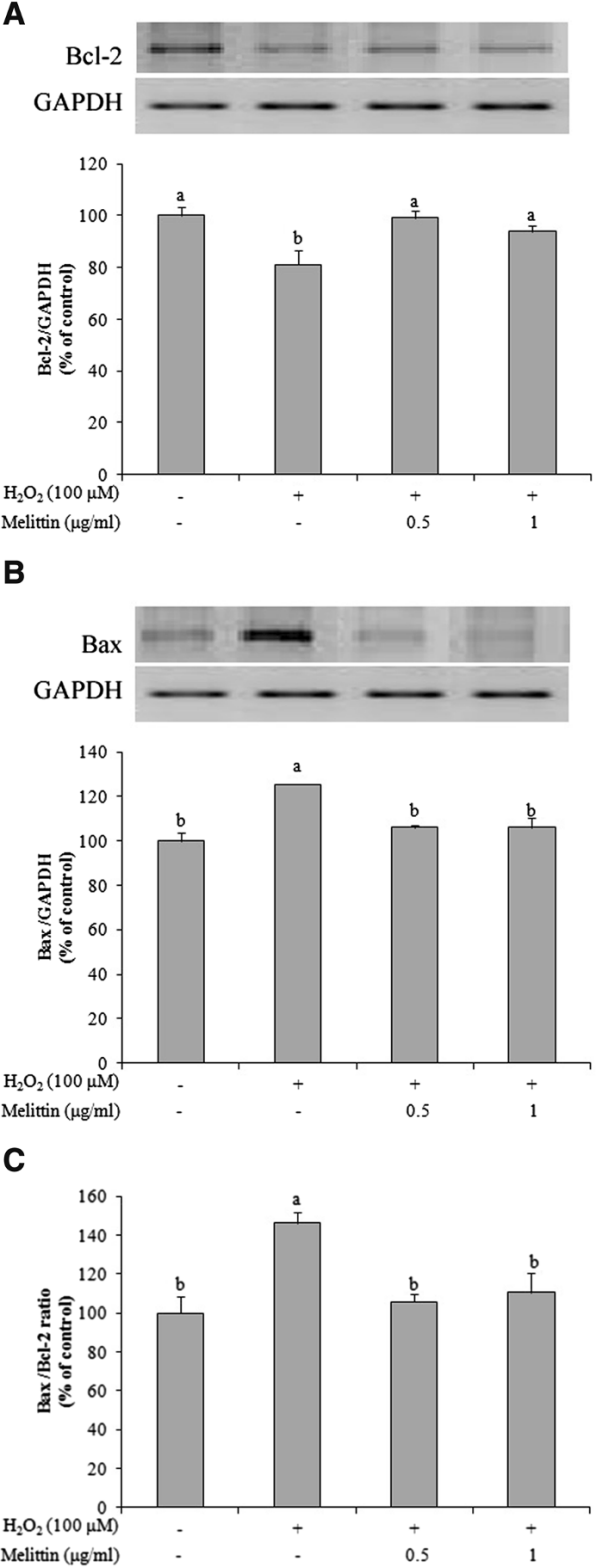
**Effect of melittin on mRNA expression of Bcl-2 and Bax in H**_**2**_ **O**_**2**_ **-treated SH-SY5Y cells. (A)** Melittin inhibited the decrease of Bcl-2 mRNA as determined by reverse transcription-PCR. **(B)** Melittin blocked the increase of Bax mRNA as measured by reverse transcription-PCR. **(C)** Melittin caused a low Bax/Bcl-2 mRNA ratio. Data are expressed as mean ± SEM from 3 independent experiments. Bars with different letters indicate significant differences at p < 0.05.

The anti-apoptotic enzyme, Bcl-2 and the pro-apoptotic enzyme, Bax play important roles in regulating cell death and cell survival [11, 12]. Therefore, we used Western blotting to investigate whether the expression of Bcl-2 and Bax protein was affected by H_2_O_2_ and/or melittin (Figure 3). In H_2_O_2_-treated SH-SY5Y cells, the expression of Bcl-2 protein was significantly decreased whereas the expression of Bax protein was significantly increased, as compared with the control, which resulted in a high Bax/Bcl-2 ratio. Pretreatment with 0.5 and 1 μg/ml melittin, however, inhibited the decrease in Bcl-2 protein and blocked the increase in Bax protein to levels similar to those of the control. In addition, melittin pretreatment led to a significantly lower Bax/Bcl-2 ratio. Next, we measured the expression of Bcl-2 and Bax mRNA using reverse transcription-PCR. Changes in the expression of Bcl-2 and Bax protein induced by H_2_O_2_ and/or melittin were due to changes in the mRNA expression (Figure 4). These findings indicate the potential of melittin to inhibit H_2_O_2_-induced SH-SY5Y apoptotic cell death.

## Discussion

Oxidative stress induces neuronal cell death, which is implicated in many neurodegenerative disorders, such as Alzheimer’s disease, Parkinson’s disease, Huntington’s disease and amyotrophic lateral sclerosis [1–3]. The underlying mechanism, however, is poorly understood. Therefore, in the present study we investigated the possible mechanism by which melittin exerts its protective effects in H_2_O_2_-induced SH-SY5Y apoptotic cell death.

Several studies have demonstrated that H_2_O_2_-induced apoptotic cell death depends on the concentration and exposure time of H_2_O_2_[21]. Gardner et al. demonstrated that moderate concentrations of H_2_O_2_ induced DNA cleavage and morphologic changes leading to apoptosis [4]. In the present study, we confirmed that SH-SY5Y cells treated with 0 to 400 μM H_2_O_2_ exhibited a dose-dependent loss of cell viability (data not shown). Pretreatment with 0.5 to 1 μg/ml melittin, however, significantly protected cell viability, which was confirmed by the lack of morphologic changes in melittin-pretreated cells. These findings suggest that melittin prevented SH-SY5Y cells from undergoing H_2_O_2_-induced apoptotic cell death. Staining the apoptotic nuclei with DAPI revealed that melittin slightly attenuated the induction of apoptotic features, such as cell shrinkage, nuclear condensation and DNA fragmentation, compared with cells treated with H_2_O_2_ alone.

Apoptotic cell death in SH-SY5Y cells induced by H_2_O_2_ is mediated by mitochondria through intrinsic pathways that activate caspases [22]. The Bcl-2 family contains two groups, an anti-apoptotic group (Bcl-2 and Bcl-x L) and a pro-apoptotic group (Bax, Bid and Bak), and these groups play a crucial role in the mitochondrial-related apoptosis pathway [23, 24]. Anti-apoptotic Bcl-2, which inhibited the release of cytochrome c, is located in the outer mitochondrial membrane [25]. In contrast, pro-apoptotic factor, Bax, resides in the cytosol and translocates to the outer mitochondrial membrane, which might lead to the loss of mitochondrial membrane potential, an increase in mitochondrial membrane permeability and the release of cytochrome c from the intermembrane space into the cytosol, leading to cell death [26]. The Bcl-2 family regulates the apoptotic process through balancing of pro-apoptotic (Bax) and anti-apoptotic (Bcl-2) products [24]. In this regard, the Bax/Bcl-2 ratio is suggested to be a useful predictor of apoptotic cell death [24, 27]. In present study, we examined the protein and mRNA expression of Bcl-2 and Bax in H_2_O_2_-induced apoptotic cell death in SH-SY5Y cells. Our findings indicate that H_2_O_2_ induced changes in the protein and mRNA expression of Bcl-2 family, Bcl-2, and Bax, in SH-SY5Y cells, but pretreatment with melittin enhanced the protein and mRNA expression of Bcl-2 and reduced the protein and mRNA expression of Bax in SH-SY5Y cells. The Bax/Bcl-2 ratio increased after treatment with only H_2_O_2_, while pretreatment with melittin inhibited the increase in the Bax/Bcl-2 ratio. These findings suggest that melittin modulates the effect of H_2_O_2_ treatment on the protein and mRNA expression of Bcl-2 and Bax.

Caspase 3 acts as an apoptotic executor by activating DNA fragmentation [28]. In apoptotic processes, cytochrome c is released from the mitochondria to the cytosol. The released cytochrome c activates caspase-9 which in turn triggers the activation of caspase-3, which induces cell death [28]. Increased caspase-3 activity is associated with an increase in the Bax/Bcl-2 ratio [26]. Although we did not examine the expression of cytochrome c and caspase-9 in the present study, our findings indicate that H_2_O_2_-induced apoptosis was associated with the expression and activation of caspase-3, which led to an increase in the Bax/Bcl-2 ratio. Pretreatment with melittin, however, inhibited the expression and activation of caspase-3, suggesting that melittin has potential anti-apoptotic effects by modulating the H_2_O_2_-induced protein and mRNA expression of Bcl-2 and Bax by downregulating caspase-3 protein expression and activation.

Melittin is a residue of the main toxic compound in honeybee venom, and is a small linear peptide composed of 26 amino acids [13, 14]. Although melittin is a toxic peptide, several studies have demonstrated various properties of melittin, including anti-bacterial, anti-arthritic, and anti-inflammatory effects [14–16]. Pratt et al. reported that melittin (2 μM) did not disrupt cell membranes of leukocytes [29]. Also, a recent study showed that a lower dose of melittin (0.5 and 1 μg/ml) protected hepatocytes against TGF-β1 [17].

## Conclusions

The findings of the present study indicate that melittin suppressed H_2_O_2_-induced apoptotic cell death in SH-SY5Y neuroblastoma cells by inducing an increase in the anti-apoptotic enzyme, Bcl-2 and a decrease in pro-apoptotic enzymes, such as Bax and caspase-3. Although further studies are needed, our results demonstrate the potential usefulness of melittin as an agent for the prevention of neurodegenerative diseases.
